# Measuring Streetscape Complexity Based on the Statistics of Local Contrast and Spatial Frequency

**DOI:** 10.1371/journal.pone.0087097

**Published:** 2014-02-03

**Authors:** André Cavalcante, Ahmed Mansouri, Lemya Kacha, Allan Kardec Barros, Yoshinori Takeuchi, Naoji Matsumoto, Noboru Ohnishi

**Affiliations:** 1 Department of Media Science, Graduate School of Information Science, Nagoya University, Nagoya-shi, Aichi-ken, Japan; 2 Department of Architecture, Nagoya Institute of Technology, Nagoya-shi, Aichi-ken, Japan; 3 Department of Electrical Engineering and Electronics, Universidade Federal do Maranhão, São Luís, Maranhão, Brazil; 4 Department of Information Systems, School of Informatics, Daido University, Nagoya-shi, Aichi-ken, Japan; MIT, United States of America

## Abstract

Streetscapes are basic urban elements which play a major role in the livability of a city. The visual complexity of streetscapes is known to influence how people behave in such built spaces. However, how and which characteristics of a visual scene influence our perception of complexity have yet to be fully understood. This study proposes a method to evaluate the complexity perceived in streetscapes based on the statistics of local contrast and spatial frequency. Here, 74 streetscape images from four cities, including daytime and nighttime scenes, were ranked for complexity by 40 participants. Image processing was then used to locally segment contrast and spatial frequency in the streetscapes. The statistics of these characteristics were extracted and later combined to form a single objective measure. The direct use of statistics revealed structural or morphological patterns in streetscapes related to the perception of complexity. Furthermore, in comparison to conventional measures of visual complexity, the proposed objective measure exhibits a higher correlation with the opinion of the participants. Also, the performance of this method is more robust regarding different time scenarios.

## Introduction

According to Rapoport, a streetscape is a more or less narrow and linear urban space lined up by buildings, used for circulation and other activities [1]. The physical and perceptual qualities of streetscapes directly influence how people interact and locally behave in the city [2], [3]. Visual complexity is one example of perceptual quality, which is related to the affective appraisal of the environment [4], [5].

In streetscapes, the interest and preference of pedestrians is shown to heavily depend on the perceived complexity [6], [7]. Specifically, pedestrians are apt to prefer streets perceived as high in complexity. Streetscape complexity is also found to influence driving behavior and performance [8]–[11]. For instance, increasing complexity normally increases the time required for reaction and peripheral detection tasks during simulated driving.

In this way, evaluation and analysis of important aspects of urban life could benefit from properly measuring or quantifying the perception of complexity in streetscapes. However, what make us perceive or decide that a visual scene “A” is more complex than a scene “B”?

Attneave showed that for scenes containing abstract shapes, certain visual characteristics (which he named symmetry, curvedness, angular variation, etc) was related to the perception of visual complexity [12]. By combining these characteristics into a single equation, Attneave created an objective measure which was correlated with human judgments on visual complexity.

The characteristics of spatial frequency have also been shown to influence the perception of visual complexity. Specifically, it is reported that the amplitude of high-frequency components must be preserved for complex objects to be recognized [13]–[15]. Similarly, specific relationships among frequency components in the phase spectrum are crucial for visual recognition of complex scenes [16]. These results have been extended by many other studies in vision research, involving many types of visual scenes.

Based on the characteristics of spatial-frequency, Näsänen et al. derived a complexity measure defined as the product between the effective image area and median frequency of the Fourier spectrum [17]. Chikhman et al. used the components of this measure to analyze complexity in hieroglyphs and contour images [18]. Notice that Näsänen's method can be applied on real-world scenes.

It is has also been shown that the presence of image edges is related to visual complexity [19]. This inspired a simple and efficient measure known as *perimeter detection*. The measurement consists of counting the number of pixels which form image edges. This procedure can be easily applied on real-world scenes by using edge-detection algorithms.

In order to measure visual clutter, a concept closely related to complexity, Rosenholtz et al. proposed a framework called feature congestion. Within this framework, several image characteristics such as contrast, color and orientation are combined into a vector space [20]. Clutter is then determined by the covariance of the space calculated at each location of the image.

Another line of research was based on the idea of computing visual complexity according to the definitions of information theory [21]. In this view, a visual scene is considered an information source, and its visual complexity is thought to be determined by the amount of information associated to its statistical distribution.

An example of information based measure is the size in bytes of the image digital file created according to coding standards such as JPEG and GIF. Theoretically, file size should increase as the amount of information increases. The JPEG file size has been used in many perception works due to its high correlation with subjective judgments of complexity. Forsythe et al. provides an extensive analysis of the performance of JPEG and also of perimeter detection [22].

Another example of information based measure is the subband entropy [23]. The subband entropy is defined as the Shannon entropy of wavelet coefficients used to encode an image.

Other methods have also been considered to evaluate visual complexity in urban environments. For instance, Elsheshtawy used a manual approach to segment meaningful elements of street houses such as windows, doorways and overall volumes of facades [24]. Complexity was then measured based on the number and variety of those elements. Cooper also used a manual technique to segment street skylines, i.e., edges formed between the boundaries of buildings and the sky [25]. Then, he used fractal dimension to assess the complexity of these skylines.

In our previous work, we have analyzed the complexity in streetscape images by using the statistics of local contrast [26]. We have found that these statistics are highly correlated with subjective judgments for daytime images. However, similar to conventional measures of complexity, they produce poor results when nighttime images are considered.

Since city streetscapes are experienced or appreciated throughout the day, proper evaluation for nighttime scenery is just as important as for those in daytime. Here, we introduce a new measure of visual complexity which exhibits a high and robust performance over different time scenarios. This measure is formed by combining the statistics of local contrast with those of local spatial frequency.

The statistics of these visual characteristics reveal structural features related to the perceived complexity in streetscapes. Specifically, subjects tend to associate higher complexity to streetscapes containing: objects which elicit high-contrast changes in their surroundings and; textures characterized by spatial frequencies lower than the average in the environment.

We conclude that while driven by different visual characteristics, the perception of complexity in streetscapes can be reliably estimated or measured by the proposed objective method.

## Methods

### Image acquisition

The streetscape ensemble consists of 74 scenes. Half of the images were acquired in Al-Kantara and Batna cities in Algeria. The other half was acquired in the cities of Kyoto and Tokyo in Japan. Within the dataset, 40 images were acquired in daytime and 34 images in nighttime.

Images were shot using the camera model Nikon D300S with lens system Nikkor AF-S DX 35 mm f/1.8G. The camera was fixed in a tripod in order to avoid artifacts due to camera shaking. Aperture and shutter speed were determined manually according to the lighting conditions in each of the 74 scenes. Image files were recorded in uncompressed color NEF format (Nikon's raw file designation). The size of the RAW images was 4288×2848 pixels and image quality was 14 bits/pixel.

### Image pre-processing for presentation

In the subjective experiments described in the next section, images were presented to participants in a 30″ display (model Dell UltraSharp 3008WFP). This display's highest resolution is 2560×1600 pixels, which prevents images being exhibited in raw size. Therefore, images were pre-processed by *decimation*. This process consists in *low-pass filtering* and then *downsampling* the image. Low-pass filtering before downsampling is performed so as to avoid *aliasing* artifacts. Here, it was used a zero-phase eighth-order low-pass Chebyshev Type I filter with normalized cutoff frequency of 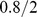. The images were then down sampled by a factor of 2. In this way, the size of the pre-processed images was 2144×1424 pixels which can be exhibited on the used display. Finally, decimated images were converted to 8 bit integer arrays so that their pixel's luminance is within the range [0, 255].

### Subjective ranking

Streetscape images were analyzed by 40 participants. Among the participants, 27 were of Japanese nationality, 13 of Algerian nationality, 25 were males, and 15 were female.

The subjects seat at a distance of approximately 80 cm from the display. Each image therefore subtended 37×25.12 degrees of visual angle. The maximum spatial frequency in an image was approximately 28.9 cycles/degree horizontally, and 28.3 cycles/degree at vertical orientation.

In order to make the subjective evaluation faster, the participants were initially asked to cluster the streetscapes into three groups: *simple*, *ordinary* and *complex*. In this regard, they were instructed to use their own perception or definition of complexity. Finally, the subjects were asked to sort images inside each group in increasing order of complexity.

**Figure 1:**
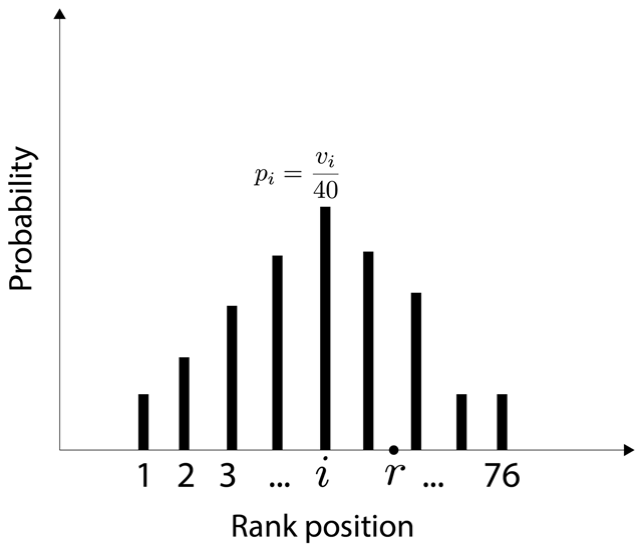
Probability distribution of rank position for one streetscape. This probability distribution describes how one specific streetscape was ranked by the participants. The 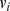 is the number of times the image was located by the subjects at position 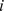. Considering 40 subjects, the probability of the image to be ranked at any specific position 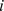 is 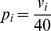. The point 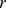 represents the mean of the distribution. Notice that there are 76 possible positions due to the two additional positions for group divisions.

After receiving the 74 ranked images from one participant, it was necessary to represent the divisions between *simple* and *ordinary*, and between *ordinary* and *complex* groups. These divisions were represented by including two additional rank positions. For example, if the group *simple* contained ten streetscapes, the division between *simple* and *ordinary* groups would occupy the 11th position in the rank. The images in the *ordinary* group would then start from position 12th. In similar manner, another additional position would be considered for the division between *ordinary* and *complex* groups. In this way, the complexity rank returned from one participant has 76 positions, which includes the 74 images plus the two group divisions. It is important to notice that images and group divisions are sorted differently by each of the 40 subjects. Thus, the rank position of a streetscape (or group division) is a random variable. The probability distribution of this variable is computed by counting the number of times 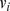 in which the image was located by the subjects at each rank position 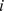. This probability distribution is represented in Figure 1.

For each streetscape, the mean 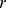 of its probability distribution of rank position is computed by using the standard definition of mean, i.e.,
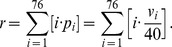
(1)Streetscapes are then finally sorted according to their mean 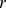. Group divisions are also included in the sorting since they also have probability distributions for rank positions. This final complexity rank is analyzed in section 3.2.

### Objective ranking

**Figure 2:**
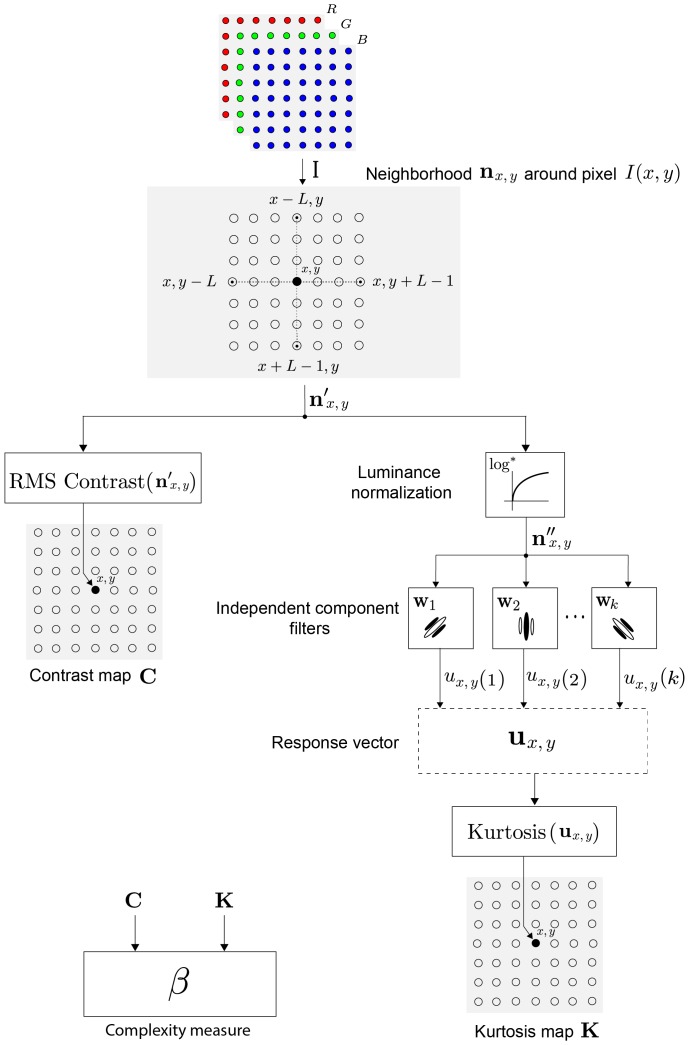
Block diagram of the objective ranking system. The RGB bands of the input streetscape are collapsed to form a grayscale image 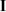. Around every pixel 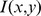, a neighborhood 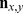 of 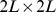 pixels is considered. After vectorization, each neighborhood 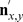 is processed by two workflows. The first workflow (left) creates a contrast map 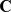 where each 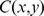 is calculated as the root-mean-squared (RMS) contrast of 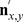. In the second workflow (right), 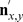 has its luminance intensities normalized by means of a log transform. Secondly, the responses of independent component (IC) filters to the normalized neighborhood form the response vector 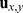. The kurtosis map 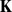 is calculated so that each 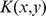 is the kurtosis value of the response 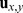. The proposed measure 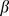 is calculated based on statistics of contrast and kurtosis maps.

The proposed measure of complexity 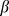 is determined by the system represented in Figure 2. The system consists of a series of image processing steps. In the first step, the RGB color bands of the input streetscape are collapsed generating the grayscale image 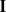. Around a pixel 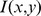 of this image, let us then consider a neighborhood 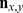 of 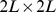 pixels.

The neighborhood 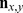 is vectorized into a column vector 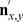, i.e.,
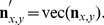
(2)The vectorization operation 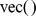 consists of reading pixels values in a column-wise fashion, i.e., from top to bottom and left to right in the neighborhood.

For all possible 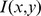, the respective 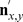 is processed by two workflows. In the first workflow (left-hand side of the block diagram), a contrast map 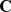 is computed based on the definition of root-mean-squared (RMS) contrast. In the second workflow (right-hand side of the block diagram), the kurtosis map 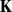 is constructed to represent spatial frequency.

The objective measure 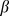 is calculated based on the statistics of the maps 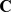 and 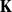. The following sections describe each part of the methodology in detail.

#### Contrast map

In the proposed system, the contrast map 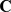 is used to highlight the local contrast in the streetscape. Each value 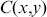 of this map is calculated as the standard deviation of vector 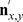, i.e.,
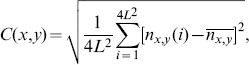
(3)where 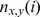 and 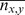 represent the 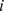-th element and the mean value of 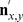. The above measure is also called the RMS contrast.

Notice that although standard RMS contrast is used, there are other definitions or measures of image contrast [27].

#### Kurtosis map

The kurtosis map 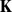 is used to segment the local spatial frequency in the scene. The computation starts by firstly log-transforming luminance values in each neighborhood, i.e.,
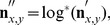
(4)where 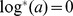 for 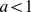 and 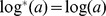 for 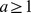. This non-linear transformation reduces large differences between luminance intensities in different parts of the image.

The spatial frequency segmentation is then carried out. The methodology is based on the concept of analyzing the response activity of high-frequency wavelet filters [28], [29]. This concept exploits the fact that such filters exhibit greater response activity for high-frequency inputs, and decreased or zero activity for low-frequency inputs. The response activity of the filters is therefore used to represent the input frequency.

In this work, the employed high-frequency wavelets are a set of IC filters, which are denoted by the vectors 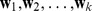. These filters are learned by the FastICA algorithm [30] from a natural scenes database. The reason why independent component analysis is used is that it automatically generates wavelet-like filters covering many orientations [31]. Furthermore, these filters are bound to be centered at high-frequencies due to second-order whitening constraints [32]. These filter properties are quantified and analyzed in section 3.3.

After the filters have been learned, each response value in vector 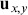 is calculated as
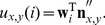
(5)Notice that in case 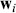 is a DC filter, its response is fixed as a constant 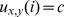 for all 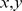.

Finally, the kurtosis map 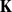 is computed as
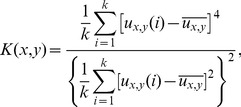
(6)where 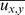 is the mean value of vector 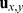. The above equation is called kurtosis and indicates either the concentration or the dispersion of probability mass away from the shoulder of a probability distribution [33]. For this reason, kurtosis has been generally used to characterize how dense or sparse is the response activity of filters [34]–[36]. Examples of how the response activity change in function of the input frequency are shown in section 3.4.

#### Measure of complexity 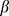


This work proposes the following objective measure to evaluate complexity:
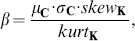
(7)where 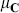 and 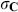 are the mean and standard deviation of contrast values 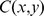, 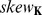 and 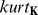 are the skewness and kurtosis of 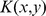 values. The relation between each of these parameters and the visual structure of a streetscape is described in the section 3.5. Basically, this measure is concerned with the presence of both high-contrast and low-frequency image regions.

## Results

### Streetscapes

**Figure 3:**
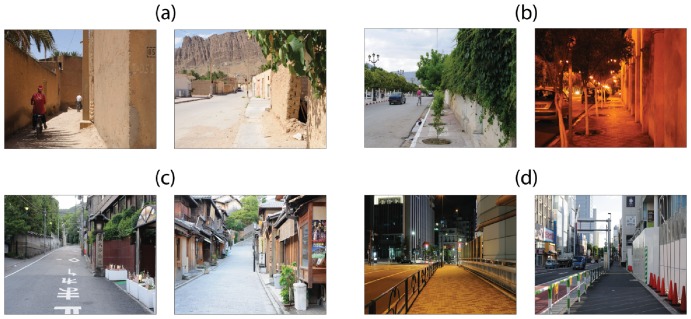
Streetscapes. (a) Al-Kantara. (b) Batna. (c) Kyoto. (d) Tokyo.

Figure 3 shows some examples of streetscapes from each city. Figure 3(a) and 3(b) show streetscapes in the Algerian cities of Al-Kantara and Batna, respectively. Figure 3(c) and 3(d) exhibit Japanese streetscapes in Kyoto and Tokyo cities.

### Subjective rank analysis

**Figure 4:**
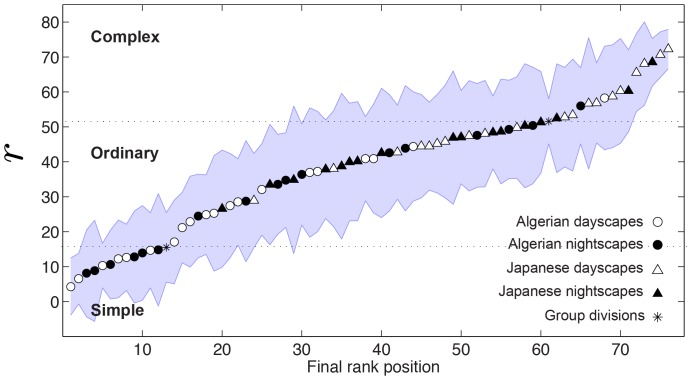
Subjective rank analysis. Streetscapes are organized in increasing order of 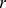-values. Circles represent Algerian streetscapes. Triangles represent Japanese streetscapes. Unfilled circles/triangles denote dayscapes. Filled circles/triangles denote nightscapes. Stars “*” represent group divisions. The blue shade represent the standard deviation around 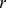.

As described in section 2.2, streetscapes are sorted according to the mean 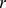 of their probability distributions of rank position. The plot in Figure 4 shows this rank. The vertical and horizontal axes give the mean 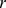 and the resulting rank position for each streetscape, respectively. The blue shade in the plot represents the standard deviation of the distributions for the streetscapes. Group divisions are also included, dividing the plot into three areas, *simple*, *ordinary* and *complex*.

It is possible to see that streetscapes in the group *ordinary* have higher standard deviation of rank position than *simple* and *complex* streetscapes. Interestingly, group divisions exhibit lower standard deviations than streetscapes.

The group *simple* consists of 12 scenes: all Algerian streetscapes; six dayscapes and six nightscapes. The category *ordinary* includes 47 scenes: 24 Algerian streetscapes and 23 Japanese streetscapes; 24 dayscapes and 23 nightscapes. The group of complex streetscape is formed by 15 images: two Algerian streetscapes and 14 Japanese streetscapes; 10 dayscapes and four nightscapes.

Algerian scenes dominate the group of simple streetscapes and the lower region of the group *ordinary*. Japanese scenes dominate the higher region of the group of ordinary streetscapes and they correspond to the great majority in the group *complex*.

In groups *simple* and *ordinary*, dayscapes and nightscapes are evenly distributed. However, dayscapes dominate the group of complex streetscapes.

Notice that the subjective rank is generated considering the entire group of 40 participants. In File S1, the subjective ranks from participants of different nationality and gender are compared. It is found that the perception of complexity is very similar for the different subgroups of subjects.

### Learned IC filters

In order to learn the set of independent component filters, a dataset of natural scenes was obtained from the McGill Calibrated Color Image Database (tabby.vision.mcgill.ca/). This database consists of TIFF formatted non-compressed images. From 100 selected scenes, 100,000 images patches of 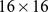 pixels were extracted in a non-overlapping fashion. This set of image patches was then used as input for the FastICA algorithm [30]. The number of learning iterations was set to 200 and the working non-linearity was the hyperbolic tangent. Dimension reduction was not used.

**Figure 5:**
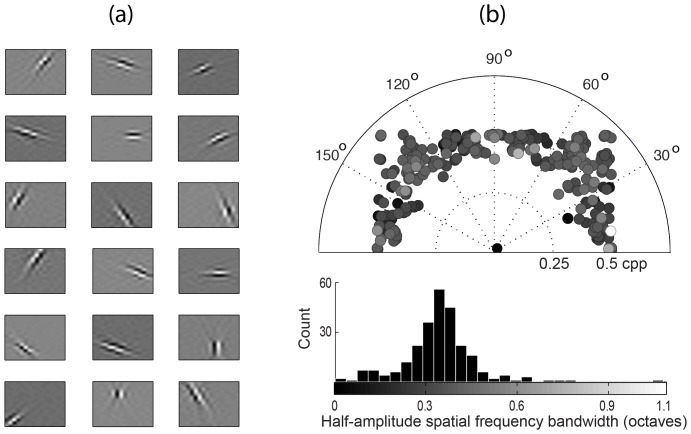
Learned IC filters. (a) Examples of learned independent component filters. (b) Filter parameters are described by the polar plot and the bandwidth histogram. Each gray-colored circle in the polar plot represents one of the filters learned by the FastICA. The distance of the circle from the origin represents the preferred spatial frequency of the filter and it is given in cycles/pixel. The orientation of the circle represents the orientation of the filter and it is given in degrees. The gray intensity of the circle represents the half-amplitude spatial frequency bandwidth. The related graymap along with the histogram of bandwidth values is shown below the polar plot.

A total of 255 filters and a DC component were learned. Figure 5(a) shows examples of the learned IC filters. The characteristics of these filters were quantified by the parameters of fitted Gabor functions. Figure 5(b) shows the parameters values.

In the polar plot, each filter is represented by a circle whose orientation and distance from the plot origin represents respectively the preferred orientation and the center spatial frequency of the filter (notice that the ICA learning process does not take into account parameters such as viewing distance, therefore, spatial frequency is given in cycles/pixel). The circle's gray intensity represents the filter's half-amplitude spatial frequency bandwidth (octaves). The associated graymap along with the histogram of bandwidths values are given below the polar plot.

The polar plot shows that the IC filters are mostly centered at the high frequency part of the Fourier spectrum. Notice the “outlier” circle in center of the polar plot which represents the DC filter. The histogram of half-amplitude bandwidth shows that the majority of filters have bandwidth values 0.3 and 0.5 octaves.

### Contrast and kurtosis maps

**Figure 6:**
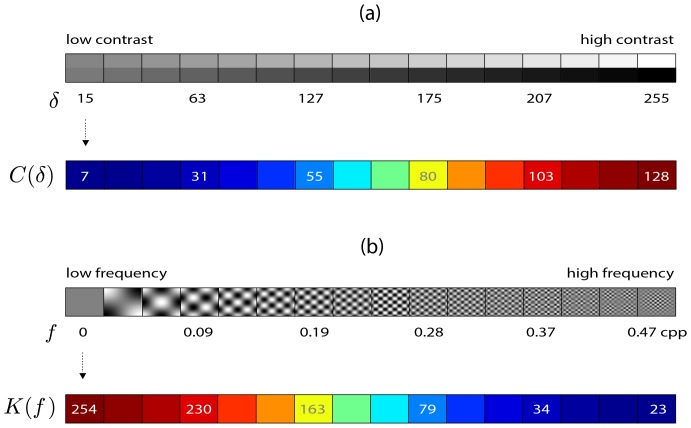
Representation of contrast and spatial frequency content by using RMS contrast and kurtosis. (a) The plot shows an array of image edges, each of 16×16 pixels. The number 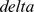 below each edge represents the luminance difference between upper and lower parts. From left to right, this luminance difference increases. The colored array of numbers 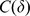 are the RMS contrast values calculated when considering each edge an image neighborhood. (b) An array of two-dimensional cosine gratings of 16×16 pixels. The number 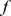 represents the spatial frequency of each grating. The colored array of numbers 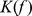 shows the respective kurtosis value generated by the proposed system.

Fig. 6 demonstrates how values in the contrast and kurtosis maps change in function of luminance difference and cycles per pixel, respectively. In Fig. 6(a), the upper plot shows an array of image edges. Each individual edge is a matrix of 16×16 pixels which contains only two luminance intensity values. Specifically, the upper half of each edge is formed by an intensity value higher than that of its lower half. The number 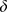 below each edge is the difference between upper and lower intensities values. From left to right in the array, the luminance difference 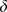 increases.

The colored array of numbers 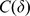 contains the respective RMS contrast values calculated for each edge (i.e., each individual edge is considered one image neighborhood, then its RMS contrast value is calculated by Eq.(3)). Colors are used to highlight low, medium and high values.

Figure 6(b) shows how kurtosis map values change. The array of control images is composed of pure two-dimensional cosine gratings of 16×16 pixels. In these gratings, horizontal and vertical components of the spatial frequency are constrained to have the same value. This frequency is represented by the number 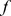 below each grating. From left to right, the frequency 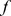 increases.

Notice that the frequency segmentation based on filter activity does not take into account viewing distance. In other words, the process is influenced only by the number of cycles per pixel and not by the number of cycles per degree. Thus, 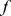 is given in cycles/pixel (cpp).

The colored array 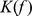 contains the respective kurtosis map values calculated when considering each grating one neighborhood (here it was used the IC filters learned in the previous section). Notice that low-frequency gratings generates high kurtosis which indicates a reduced response activity from the IC filters. High-frequency gratings, however, generate low kurtosis values indicating a dense filter response activity.

**Figure 7:**
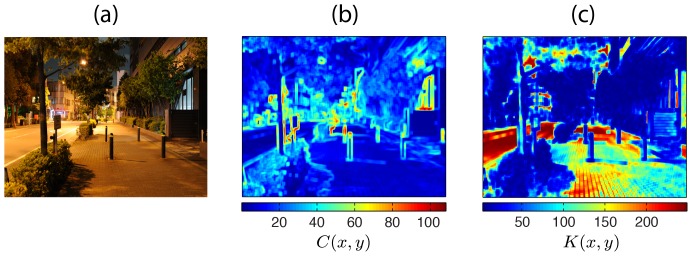
Contrast and kurtosis maps. (a) Original image. (b) Respective maps 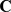 and (c) 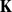. Colormaps associated with RMS contrast and kurtosis values are shown below each map.

In Fig. 7, true contrast and kurtosis maps are exhibited for an example of streetscape image. These maps were calculated using neighborhoods of 16×16 pixels. In the streetscape, objects which luminance intensities contrast with their surroundings generate high values in the contrast map. One can notice, however, that most of the structures present in the scene do not generate such high values of contrast.

In the kurtosis map, low-frequency areas such as the road generate high kurtosis values. On the other hand, textured regions such as the vegetation and the sidewalk have higher energy in high-frequencies generating lower kurtosis values.

### Statistics of contrast and kurtosis maps

**Figure 8:**
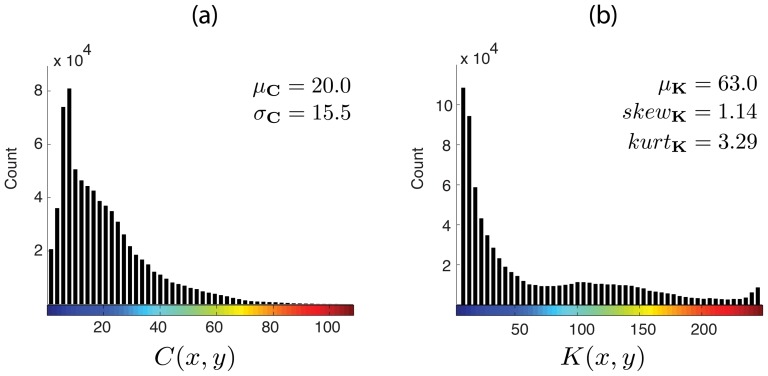
Statistics of contrast and kurtosis maps. (a) Histogram of the contrast map in Figure 7. (b) Histogram of the kurtosis map in Figure 7. The statistics of these p.d.f.s are presented at the top-right corner of the histograms. Colormaps for 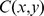 and 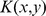 values are preserved for easy understanding.

Figure 8 shows the histograms of the contrast and kurtosis maps exhibited previously in Fig. 7(b) and (c). By using these histograms, one can analyze more precisely the distribution of local contrast and spatial frequency within the streetscape in Fig. 7(a).

For instance, in Fig. 8(a), the histogram of the contrast map shows more clearly the number of low-contrast locations in relation to that of high-contrast. However, while the maps and their histograms are useful for visual inspection and interpretation of the streetscape structure, they are not simple quantities. In other words, they can not be used directly as objective measures of the visual attributes of the streetscape.

The statistics of the maps on the other hand are quantities which describe very specific characteristics of the streetscape. Fig. 8 shows the statistics of the contrast and kurtosis maps which are used in the proposed measure of complexity 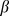.

The first statistic is the mean 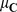 of contrast values 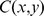, which is by definition a positive number. The mean value 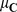 increases as the number of high-contrast regions increases.

The second statistics is the standard deviation 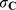 of contrast values 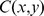. 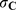 can increase due to two factors. Firstly, it increases in the presence of image regions that generate contrast values 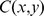 higher than mean 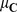. On the other hand, it also increases with regions that yields contrast values lower than 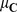. In this way, 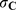 represents the contrast “variety” in the streetscape image.

Regarding the kurtosis map, two statistics are used in measure 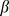: the skewness 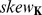 and the kurtosis 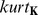 of 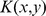 values (one must not confuse 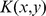 values with the kurtosis 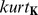 of their distribution).

Both skewness and kurtosis depend on the mean 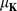 of the 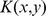 distribution. The skewness is generally regarded as a measure of asymmetry of a distribution in relation to its mean. For instance, if there is a tendency for 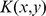 values to be higher than the mean 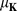 (i.e., the distribution is asymmetric towards its right-hand tail), then the skewness of the distribution is positive. On the other hand, in case distribution values tend to be lower than the mean, then skewness is negative. If the probability density distribution is symmetrical around its mean, the skewness is zero.

In Fig. 8(b), the positive skewness, 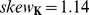, indicates asymmetry towards 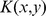 values higher than the mean 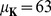. Notice that higher 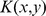 values represent lower frequencies. Therefore, this positive 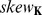 indicates asymmetry towards low-frequencies. In other words, there is a significant number of streetscape regions characterized by spatial frequencies lower than that represented by the mean 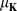.

For highly skewed distributions, however, it is important to investigate the presence of statistical outliers. These are generally defined as values *extremely* higher or lower than the mean of the distribution. For instance, in case of the histogram in Fig. 8(b) with mean 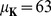, outliers would be located at the extreme of the right-hand tail of the distribution.

Due to the properties of kurtosis, the magnitude of 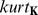 heavily reflects the presence of such values. Thus, 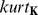 is used in the denominator of measure 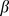 to compensate 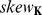 values which are high due to outliers in the 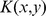 distribution.

**Figure 9:**
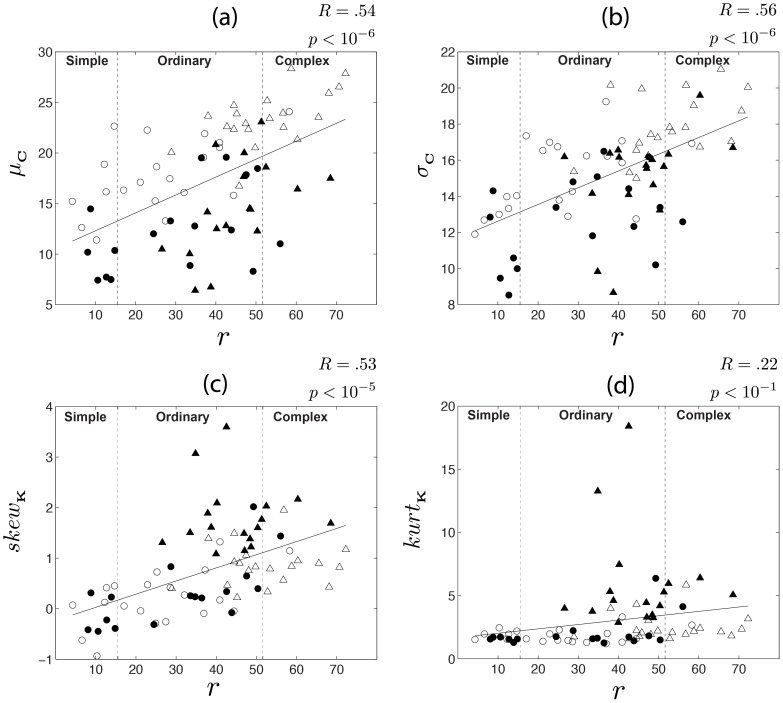
Statistics of contrast and kurtosis maps (cont.). Statistics are given in function of the subjective rank 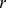. (a) Mean contrast 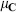. (b) Standard deviation 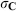 of contrast values. (c) Skewness of 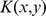 values. (d) Kurtosis of 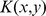 values. Correlation coefficient between objective and subjective ranks are given at the top right corner of each plot. Vertical dotted lines represents the divisions between categories *simple*, *ordinary* and *complex*. In each scatter plot, the solid line represents the best least-squares-sense first-order polynomial fit. The numbers 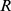 and 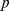 at the top-right corner of each plot indicate the Pearson's correlation coefficient and its p-value, respectively.

Figure 9 shows how the statistics of the contrast and kurtosis maps correlate with the subjective complexity rank 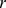. In the scatter plot 9(a), the mean contrast 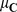 is given in function of 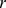-values. The correlation coefficient between 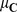 and the subjective rank is R = 0.56.

The plot 9(b) shows the statistic 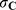. The correlation coefficient between 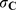 and 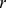 is R = 0.57. Notice that the majority of nightscapes present lower 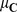 and 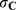 than dayscapes.

The positive correlations between 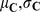 and the subjective rank indicate that complex streetscapes exhibit a higher number of objects or structures which elicit high changes of luminance and contrast in the scene.

The scatter plot 9(c) shows 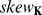. This statistics has a correlation coefficient of R = 0.53 (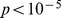) with the subjective rank. This shows that the number of regions characterized by spatial frequencies lower than the mean in the streetscape tend to increase with complexity.

Fig. 9(d) shows 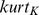. The correlation coefficient between 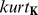 and the subjective rank is R = 0.22, with a high 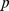-value. This indicates that these variables are not significantly correlated. However, 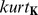 is an important statistic since it signalizes outliers in the 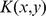 distributions of the streetscapes.

The proposed measure 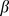 is built as a direct combination of these observations on the characteristics of contrast and spatial frequency of streetscape scenes.

### Objective rank analysis

This section shows how 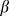 correlates with the subjective rank 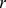 given in Fig. 4. Here, the following conventional measures are also analyzed: perimeter length, JPEG file size, subband entropy, feature congestion and Näsänen's measure.

**Figure 10:**
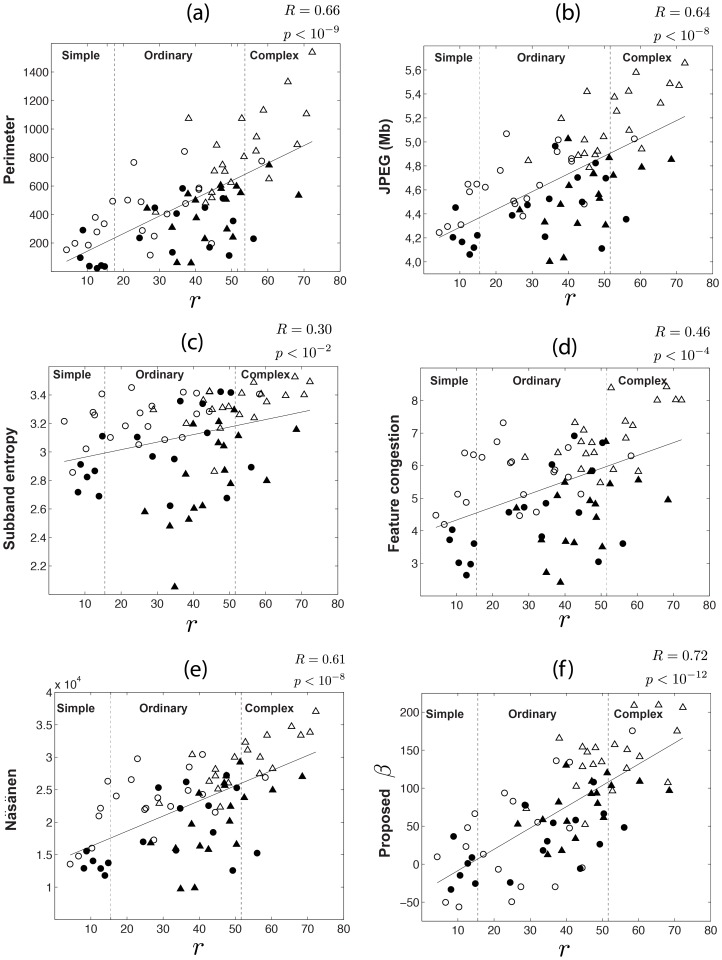
Correlation between objective measures and subjective rank. Objective measures are given in function of subjective rank 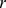. (a) Perimeter length. (b) JPEG file size given in *megabytes*. (c) Subband entropy. (d) Feature Congestion. (e) Nasanen. (f) Measure 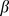. Correlation coefficient between objective and subjective ranks are given at the top right corner of each plot. Vertical dotted lines represents the divisions between categories *simple*, *ordinary* and *complex*. In each scatter plot, the solid line represents the best least-squares-sense first-order polynomial fit.

Scatter plots in Fig. 10 present the correlation behavior of the measures over the entire streetscape dataset. Figures 10(a) and 10(b) show the behavior of perimeter length and JPEG file size (see File S1 for parameter settings descriptions). These measures exhibit similar correlation coefficients with the subjective rank. In the scatter plots of both measures, nightscapes consistently receive lower values than dayscapes.

Figures 10(c) and 10(d) exhibit measures subband entropy and feature congestion. Fig. 10(e) and 10(f) shows the behavior of Nasanen's and the proposed 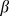. Notice that although these measures exhibit quite different correlation coefficients, they are also seem biased by nightscapes in same sense of the previous measures. Still, the proposed 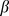 exhibits the highest correlation when all streetscapes are considered (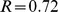).

Table 1 exhibits the correlation coefficients when streetscape types are considered separately. From Table 1, it is clear that all objective measures have higher performance for daytime images. For instance, the correlation coefficient of JPEG file size is 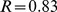 for dayscapes and only 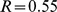 for nightscapes. On the other hand, the proposed 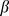 exhibits the highest correlation for nightscapes, i.e., 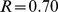. Notice that 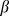 also has the least variability between the correlation coefficients for dayscapes and nightscapes.

**Table 1:** Correlation coefficients between objective and subjective ranks for individual streetscape types.

	Perimeter	JPEG	SB Entropy	FC	Näsänen	Proposed 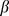
Dayscapes	**0.79**	**0.83**	**0.53**	**0.65**	**0.80**	**0.77**
Nightscapes	0.59	**0.55**	0.27	0.46	**0.57**	**0.70**
Japan	**0.66**	**0.63**	**0.56**	**0.56**	**0.71**	**0.60**
Algeria	0.41	0.44	0.30	0.27	0.42	**0.51**
Simple	0.48	0.52	0.30	0.45	0.65	0.25
Ordinary	0.18	0.21	0.12	0.1	0.19	0.41
Complex	0.45	0.39	0.31	0.36	0.50	0.36

Correlation coefficients are calculated considering only the number of images in the specific streetscape type referenced in the most left column. Values in **bold** font represents significant correlation coefficients (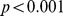).

There are also variations in correlation for the other types of streetscapes. For instance, objective measures exhibit higher correlation coefficients for Japanese scenes than for those from Algeria. In this case, the proposed 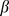 also exhibits less variation than other measures. For simple and complex scenes, Näsänen's measure is the most correlated with the subjective rank, i.e., 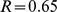 and 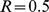, respectively. For ordinary category, 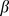 has the highest correlation 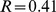. Notice that for these three categories, 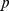-values are higher than 0.001.

## Discussion

Much has been understood about how the early visual system responds to contrast and spatial frequency. And while there is no established model of how these early responses influence the perception of complexity, it is interesting to consider physiological results that are related to 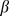 (notice that for primitive shapes, response activity of visual cells increases with complexity [37]).

Local contrast can vary significantly within a visual scene [38]. A contrast map is a easy way to visualize this variation in terms of lower and higher contrast image areas. Now from a physiological point of view, it is important to understand how the response of early visual cells is influenced by these low and high contrast areas. Many studies have reported that in general firing rate of visual cells is not linearly related to the input contrast [39]–[41]. Specifically, firing rate increases linearly with contrast but reaches saturation at high contrast values. Furthermore, there are thresholds or contrast below which cells do not respond.

Measure 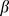 is linearly related to the mean contrast of the streetscapes. Therefore, it increases with contrast but does not saturate as in the case of cell firing rate. Also, it does not account for any threshold effects. In order to mimic the physiological behavior, a proper non-linear transform would have to be applied on the contrast map in order to threshold and saturate contrast values.

The contrast sensitivity function (CSF) is another important physiological result that is related to the perception of spatial frequency [13]. The CSF defines how much contrast is need to perceive a spatial-frequency component. While the CSF can be different for each person, it generally shows that low-frequency components requires lower contrast to be perceived than high-frequency components. In other words, CSF shows that human subjects have higher sensitivity for low frequencies. Notice however that adaptation and masking effects during natural vision reduce this sensitivity after some period of exposure [42].

The contrast and kurtosis maps provide estimations of contrast and spatial frequency for each region within a scene. According to the CSF, spatial frequency components cannot be perceived in case image regions do not have the required minimum contrast. Since the current methodology does not account for the CSF, the frequency estimations for each image region may differ from what is actually perceived.

The discrepancy between what is measured and what is perceived could be significant specially for nighttime images due to lower luminance and contrast. In fact, it is known that visual acuity (i.e., the maximum perceived spatial frequency) is reduced in low luminance scenes [43]. Furthermore, changes in eye optics due to low luminance can introduce aberrations. These aberrations have the effect of decreasing the transmitted contrast for medium and high-spatial frequencies [44].

Changes in the distribution of light from daytime to nighttime also heavily influence the perception and interpretation of the “architectural” space [45]. Specifically, it is found dim light often results in shrinking the perceived size of objects, ornaments and the overall built environment. Unaccounted factors related to perception in low luminance and contrast might be the reason for the degraded performance of complexity measures in nightscapes.

The above are just few examples of issues related to the physiological processing and perception of contrast and spatial frequency. Notice that some of the complexity measures do not directly exploit these image properties. However, the characteristics of contrast and spatial frequency do influence the measurements in those methods. Furthermore, these methods are also strongly supported on knowledge about the early visual system.

The perimeter detection method, for example, is based on the number of edges detected in the scene (see File S1). The process of edge detection is closely related to the filtering performed by the *simple* cells of the primary visual cortex (V1) [46], [47]. Specifically, these cells have very dedicated or specialized receptive fields. Due to this characteristic, simple cells have been primarily viewed as biological edge detectors [48], [49]. According to this, one could associate the perimeter detection measure to the activation of simple cells.

Further research, however, shown that the characteristics of V1 receptive fields could be artificially generated by *efficiently encoding* natural scenes [50]. In this coding process, filters are generated according to optimization functions which goal is to maximize the amount of information extracted from the input signal. These results support a broader view of V1 cells where they are adapted to efficiently encode visual stimuli found in the environment [34].

Due to the nature of the signal filtering performed in the subband entropy method and in coding schemes such as JPEG, Rosenholtz suggests that these systems are likely to capture some of same information that is extracted by V1 cells [23]. Notice that the methodology used to compute our kurtosis map is also a V1-like filtering technique. However, in contrast to the subband entropy and JPEG filtering, the independent component filters strongly focus on high-frequency bands.

In regard of JPEG filtering, it is also worthy noticing that there are additional constraints which are inspired by the human visual system. Specifically, the loss of information during coding is controlled so that low-frequency image components suffer less losses than high-frequency components. This rationale is derived from the human contrast sensitivity function.

After an image has been encoded by JPEG, the size of the digital file may be thought as the amount of information left in the image after losses. Similar thinking can be used to interpret the subband entropy measure. In this case, entropy represents the total amount of information in the frequency bands since there are no losses involved.

Interestingly, it has been shown that the size of the JPEG file is highly correlated with the number of edges in an image (i.e., the perimeter length measure) [22]. This result corroborates the connection between edge detection and coding of visual scenes. The correspondence between JPEG file size and the perimeter length measure can also be observed for streetscapes. As shown in Fig. 10, these measures have quite close correlation coefficients with the subjective rank. Even analyzing at a streetscape type level (see Table 1), the maximum difference between their coefficients is not higher than 0.05. It is easy to see that this does not hold for any other pair of measures.

In this way, the measures of visual complexity analyzed here share similarities in terms of physiological foundations, image processing methodology, and correlation behavior with the subjective rank. In summary, methods employ filtering techniques to extract low-level image characteristics which have well-understood influence in the human visual system. The objective measures are then derived in function of a single or a combination of these image characteristics.

Our results suggests that low-level image characteristics are indeed related to the complexity perceived in streetscapes. On daytime images for example, the use of these characteristics allow objective measures to be highly correlated with the opinion of participants. Nonetheless, the effectiveness of these methods can considerably fluctuate across streetscape types. The same behavior is noticed for categories of images different than streetscapes [18], [22]. These studies suggest that different low-level characteristics may best suit different image categories.

In case of streetscapes, the statistics of local contrast and spatial frequency provide a competitive performance in comparison to the state-of-art methods. In fact, considering the entire dataset, the proposed measure 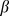 exhibits the highest correlation with the subjective rank.

Measure 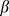 has also less variability in correlation with subjective perception from daytime to nighttime images. For streetscapes, this is an important advantage. For instance, a more stable measure could be used to analyze visual interest and preference of pedestrians without requiring changes in the methodology. Furthermore, it could be used to analyze the human perception during nighttime driving, which is has been pointed out as a difficult problem since the visual system behaves differently from daytime to nighttime [51].

The proposed measure also provides insight on the morphological features of the built space which are related to the perception of complexity. Specifically, in streetscapes high complexity is found correlated with the presence of high contrast structures and areas defined by spatial frequencies lower than the average in the scene. High contrast image features and the energy in low frequencies are in fact reported to drive human attention or emotional event processing.

Now, the definition of streetscapes given in the introductory section clearly indicates that this category can hold very heterogeneous scenes. Diversity can come from many factors such as different types of architecture, geography, time scenario, and even season which directly influence the city vegetation.

Therefore, objective measures based on reduced sets of low-level image characteristics are unlikely to be satisfactory for all possible streetscapes. The statistical framework proposed in this work can be easily applied to identify new image characteristics related to the perception of complexity.

The diversity in this category also suggests that different perceptual mechanisms may engage during subjective evaluation of different streetscapes. As discussed before, the methods are still quite limited in accounting for such mechanisms. A proper implementation of perceptual related processes could improve objective measures with higher and more stable performance across different types of streetscapes.

## Conclusion

The complexity perceived in streetscapes is known to influence important elements in urban life such as the visual interest of pedestrians and driving behavior. In this work, a methodology is proposed for objectively measuring streetscape complexity based on the statistics of local contrast and spatial frequency. The proposed method exhibits higher correlations with subjective perception in comparison to conventional measures of complexity. Furthermore, it is found that this method is more effective and robust for nighttime scenes.

The proposed method also revealed structural features in streetscapes related to the perception of complexity. Specifically, it is found that higher complexity is associated with the presence of high contrast objects and image areas characterized by spatial frequencies lower that the average in the environment.

Since complexity can be related to different features in streetscapes, future studies will investigate the influence of different image characteristics and the effect of implementing physiological mechanisms related to human perception.

## Supporting Information

File S1 Supporting material.
(PDF)
