# Supplementary material for: Measuring Streetscape Complexity Based on the Statistics of Local Contrast and Spatial Frequency
Source: PLoS One. 2014 Feb 3;9(2):e87097. doi: 10.1371/journal.pone.0087097 (PMC3911930; doi:10.1371/journal.pone.0087097)
Supplement: File S1 — Supporting material. (PDF) [file pone.0087097.s001.pdf]

Supporting material for:

Measuring streetscape complexity based on the statistics of local contrast and spatial frequency

André Cavalcante[1,*], Ahmed Mansouri[2], Lemya Kacha[2], Allan Kardec Barros[3], Yoshinori Takeuchi[4], Naoji Matsumoto[2], Noboru Ohnishi[1]
1 Department of Media Science, Graduate School of Information Science, Nagoya University, Nagoya-shi, Aichi-ken, Japan
2 Department of Architecture, Nagoya Institute of Technology, Nagoya-shi, Aichi-ken, Japan
3 Department of Electrical Engineering and Electronics, Universidade Federal do Maranhão, São Luís, Maranhão, Brazil
4 Department of Information Systems, School of Informatics, Daido University, Nagoya-shi, Aichi-ken, Japan
∗ **E-mail: andre@ohnishi.m.is.nagoya-u.ac.jp**

Subjective ranks for subgroups of participants

Figure S1 shows the subjective ranks computed considering subgroups of participants divided by nationality and gender. The table S1 shows the correlation coefficients between the subjective ranks of the distinct subgroups.
Table S1 shows that the perceptions annotated from different subgroups of participants are significantly correlated. This indicates that the Algerian and Japanese subjects have very similar opinions about the complexity perceived in the streetscape dataset. This is also true in case of male and female participants. However, it is clear that the influence of nationality is stronger than that of gender. Specifically, the correlation coefficient between the subjective rank from males and that from females is very high, i.e., R=0.97.

Parameter settings

The methods used for comparison (i.e., perimeter length, JPEG file size, subband entropy and feature congestion) are based on image processing flows which require input settings. In order to determine these settings, an analysis is carried out for each method. Specifically, input settings are varied so as to maximize the correlation of objective measures with the subjective complexity rank. The methods are only considered for comparison when using settings which generates the highest correlation. It also is important to notice that some of these methods are not based on multi-scale processing. Thus, the input size of the images may influence their performance. In order to verify any effect regarding this issue, additional decimation steps are included at the beginning of the processing flows. These decimation steps have the function of modifying the input size of the images before computation. The performance of the methods are then analyzed over several input sizes.
Firstly, the perimeter length is discussed. In this method, the objective complexity measure is the number of pixels which belong to image edges. Here, Roberts algorithm is used to detect edges in the image. This algorithm detects edges by checking whether the gradient of pixel values is higher than a chosen threshold. The threshold value is an input setting for perimeter length and should be chosen before the method is used. Thus, it is important to analyze the behavior of the method over many threshold values. Since this method is not based on multi-scale processing, it is also interesting to analyze the effect of image size by using decimation.

In Figure S2(a), the correlation coefficient between the objective measure and the subjective rank is given in function of the threshold value and image size. The correlation increases as the threshold value increases. It is also easy to see that reducing the size the of images before edge detection increased the correlation. The settings which generates the highest correlation ($R = 0.66$) is image size of 268 x 178 for a threshold value of 62. This is indicated in the plot by an arrow.
For the JPEG method, the objective complexity measure is the size of the digital image file. In the JPEG coding process, the input data is decomposed in several components through a transformation called *Discrete Cosine Transform*. After this decomposition, each resulting component is processed by *quantization*. Normally, this step consists of a non-linear transformation which decreases the resolution of the amplitude values of the components.
In standard JPEG, there is parameter called quality factor Q which controls the overall loss of information during the quantization step. Factor Q can assume values in range $[1, 100]$, where higher values indicates files with higher visual quality.
For each Q, it is calculated here the correlation coefficient between the resulting JPEG file sizes and the subjective rank. Figure S2(b) shows how the correlation depends on the parameter Q and image size. The correlation coefficient is higher for low values of quality factor. Similar to perimeter length, the correlation also increases as the image size is reduced. Interestingly, the plot shows that the effect of Q on the correlation diminishes as the image size is reduced. The JPEG settings which generates the highest correlation ($R = 0.64$) are $Q = 1$ and image size of 536 x 356 pixels. In the subband entropy, the objective measure is the Shannon entropy of wavelet coefficients. The method start by firstly converting images into CIELab space. Then it calculates wavelets coefficients from luminance and chrominance channels in different scales. Finally, it sums the entropies of the coefficients from each scale. Figure S2(c) shows how the correlation coefficient between this sum and the subjective rank depend on the number of scales and image size. The highest correlation ($R = 0.30$) is generated for a number of three scales and image size 268 x 178 pixels.
In feature congestion, the objective measure is computed as the volume of the covariance of a vector space constructed by "features" such as color, contrast and orientation. Feature congestion uses several setting parameters such as number of scales over which the covariance is computed, width of several types of filters, etc. In total, the performance of feature congestion method is analyzed here over five setting parameters. Since it is not straightforward to graphically represent a 5-D function, Figure S2(d) exhibits only the best result found for each image size after varying the settings. Over all images sizes, 536 x 356 pixels generates the highest correlation coefficient ($R = 0.46$) for feature congestion.
Nasanen's objective measure is defined as the product between the median spatial frequency of the image energy spectrum distribution and the image area which comprises the 95% of the total image energy. Figure S2(e) exhibits the correlation of Nasanen's measure with subjective rank in function of image size. Notice that the image area is also varied for several energy thresholds. The highest correlation is found for image size 134 x 89 pixels and area comprising 80% of the total image energy.
Finally, Figure S2(f) gives the correlation of the proposed β in function of image size. The highest correlation ($R = 0.72$) is found for image size of 1072 x 712 pixels.

Table S1. Correlation coefficients between subjective ranks computed from subgroups of participants. Values in **bold** font represents significant correlation coefficients ($p < 0.001$).

	Correlation
Algerian x Japanese	**0.87**
Males x females	**0.97**

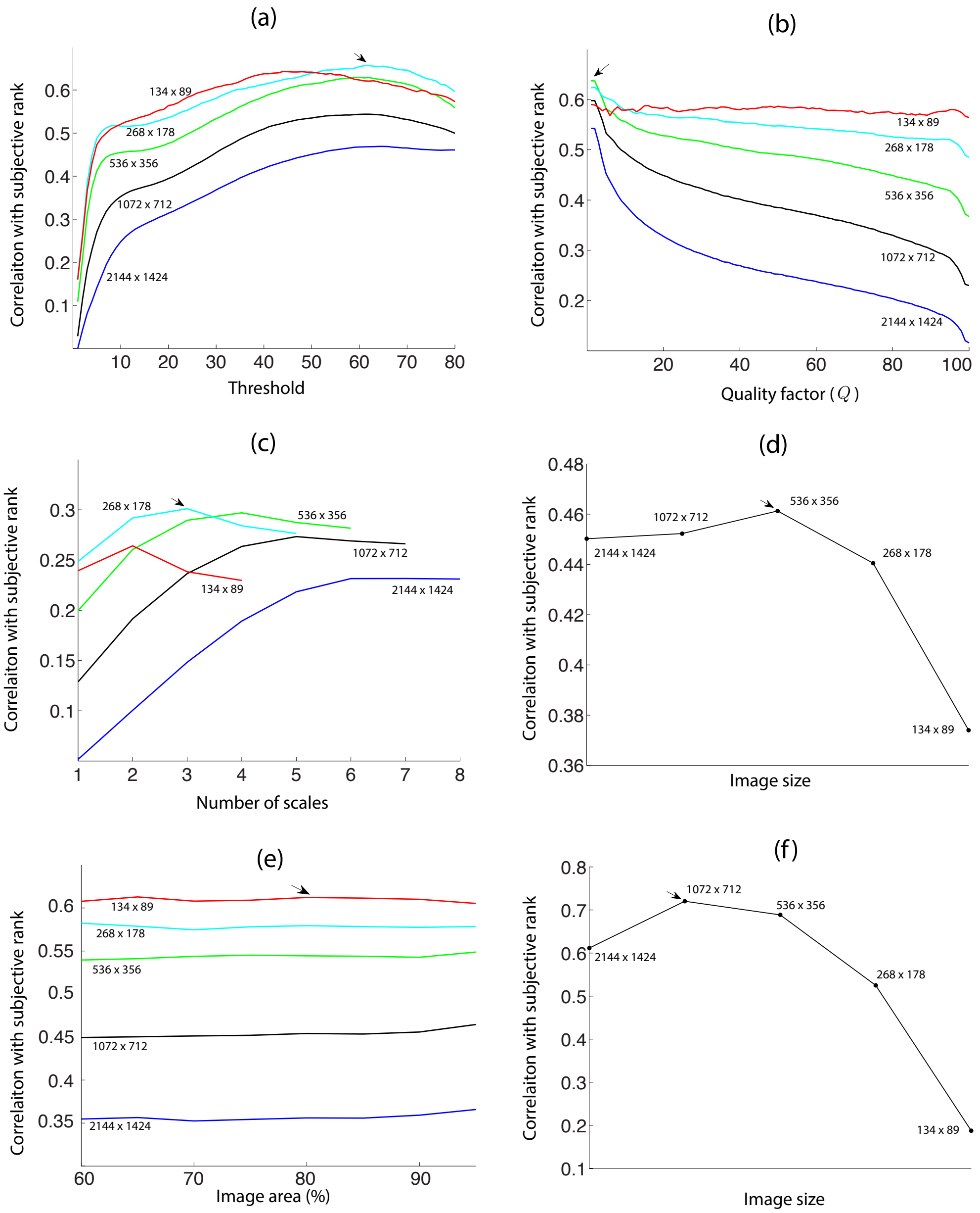

Figure S1. Correlation with subjective rank in function of settings. Correlation coefficient between objective measures and the subjective rank is given in function of setting parameters. (a) Perimeter length. (b) JPEG. (c) Subband entropy. (d) Feature congestion. (e) Näsänen. (f) Proposed β. Settings which generates the highest correlation coefficients are indicated by an arrow.

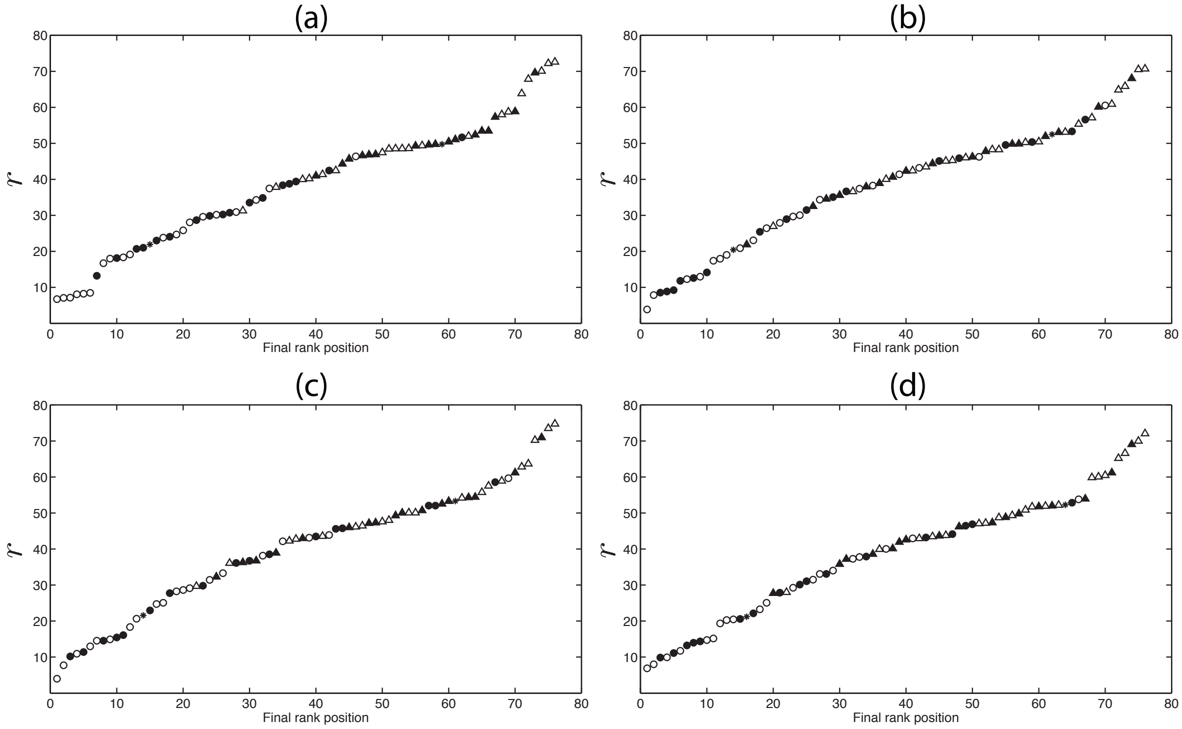

Figure S2. Subjective ranks generated for subgroups of participants. (a) Algerian participants. (b) Japanese participants. (c) Males. (b) Females.
